# Access to device-aided therapies in advanced Parkinson’s disease: navigating clinician biases, patient preference, and prognostic uncertainty

**DOI:** 10.1007/s00702-023-02668-9

**Published:** 2023-07-12

**Authors:** Manon Auffret, Daniel Weiss, Fabrizio Stocchi, Marc Vérin, Wolfgang H. Jost

**Affiliations:** 1 France Développement Electronique (FDE), Monswiller, France; 2 Institut des Neurosciences Cliniques de Rennes (INCR), Rennes, France; 3 Behavior and Basal Ganglia Research Unit, CIC-IT, CIC1414, Pontchaillou University Hospital and University of Rennes, Rennes, France; 4 Centre for Neurology, Department for Neurodegenerative Diseases, Hertie-Institute for Clinical Brain Research, Hoppe-Seyler-Str. 3, 72076 Tübingen, Germany; 5 University San Raffaele Roma and Institute of Research and Medical Care IRCCS San Raffaele Roma, Rome, Italy; 6 Neurology Department, Pontchaillou University Hospital, rue Henri Le Guilloux, 35000 Rennes, France; 7 Parkinson-Klinik Ortenau, Kreuzbergstr. 12-16, 77709 Wolfach, Germany

**Keywords:** Parkinson’s disease, Device-aided therapies, Clinician bias, Personalised medicine, Patient preference

## Abstract

Device-aided therapies (DAT), which include deep brain stimulation and pump-based continuous dopaminergic stimulation with either levodopa or apomorphine, are among the major advances in the clinical management of Parkinson’s disease (PD). Although DAT are being increasingly offered earlier in the disease course, their classical indication remains advanced PD. Theoretically, every patient should be offered transition to DAT when faced with refractory motor and nonmotor fluctuations and functional decline. Worldwide clinical reality is far from these ideal, and, therefore, question the “real-world” equal opportunity of access to DAT for PD patients with advanced PD—even within a single health care system. Differences in access to care, referral pattern (timing and frequency), as well as physician biases (unconscious/implicit or conscious/explicit bias), and patients’ preferences or health-seeking behaviour are to be considered. Compared to DBS, little information is available concerning infusion therapies, as well as neurologists’ and patients’ attitudes towards them. This viewpoint aims to be thought-provoking and to assist clinicians in moving through the process of DAT selection, by including in their decision algorithm their own biases, patient perspective, ethical concerns as well as the current unknowns surrounding PD prognosis and DAT-related long-term side effects for a given patient.

## Introduction

“*The existing guidance tends to imply that right answers exist rather than recognising the complex trade-offs that have to be made between conflicting aims.”*

Barber BMJ. 1995 Apr 8; 310(6984): 923–925.

Device-aided therapies (DAT), which include brain surgery and pump-based continuous dopaminergic stimulation (with either levodopa or apomorphine), are among the major advances in the clinical management of Parkinson’s disease (PD) (Krüger et al. 2016; Obeso et al. 2022). Stemming from brilliant empirical clinical experiments led in the 1970s and having entered clinical practice in the late 1990s–early 2000s (Table 1), they have revolutionised treatment options and patients’ quality of life. Their development is intertwined with the understanding of PD pathophysiology and, more broadly, of ever-growing technological advances (neurosurgery, pump technology). Four DAT are currently available in many parts of the world: (1) deep brain stimulation (DBS) of different targets (subthalamic nucleus—STN and globus pallidus internus—Gpi to treat dopaminergic fluctuations, and ventral intermediate nucleus of the thalamus—VIM for parkinsonian and non-parkinsonian tremor), (2) continuous subcutaneous apomorphine infusion (CSAI), (3) levodopa–carbidopa intestinal gel (LCIG), also designated in the United States of America (USA) as carbidopa–levodopa enteral suspension (CLES) (Burack et al. 2018), and (4) levodopa–entacapone–carbidopa intestinal gel (LECIG).

**Table 1:** Landmarks in the history of DAT in PD

Date	Authors/country	DAT	Discovery/event
1975	Bechtereva et al. (Russia)	Pre-DBS	*Electrical stimulation of the brain subcortical structures* in the treatment of chronic diseases of the nervous system, including hyperkinesis in parkinsonism
	Shoulson et al. (USA)	Pre-LCIG	*Intravenous levodopa administration* in PD patients with on–off response
1982	Quinn et al. (UK)	Pre-LCIG	Intravenous infusion of levodopa in PD patients experiencing fluctuations: *« perhaps the ****continuous intragastric infusion of levodopa**** might produce similar results»*
1986	Obeso et al. (Spain)	Pre-CSAI	Continuous subcutaneous administration of lisuride (portable mini-infusion pump)
	Ruggieri et al. (Italy)		
	Castro-Caldas et al. (Portugal)		
1987	Benabid et al. (France)	DBS	Combined (thalamotomy and *stimulation*) stereotactic surgery of the thalamic nucleus ventralis intermedius (VIM) for bilateral PD
	Obeso et al. (Spain)	CSAI	*Subcutaneous infusion of apomorphine* in the treatment of PD fluctuations
1988	Stibe et al. (UK)	CSAI	
1989	Sage et al. (Sweden)	LCIG	Continuous *enteral levodopa infusions* in advanced PD
	Ruggieri et al. (Italy)		Jejunal delivery of levodopa methyl ester
1991	Benabid et al. (France)	DBS	*Thalamic stimulation* for the treatment of tremor (PD and other movement disorders)
	Blond & Siegfried (France, Switzerland)		
1993	Bredberg et al. (Sweden)	LCIG	Intraduodenal infusion of levodopa, *nasoduodenally* delivered by a *portable pump*
1994	Siegfried & Lippitz (Switzerland)	DBS	Bilateral chronic electrostimulation of ventroposterolateral *pallidum* in PD
1995	Limousin et al. (France)	DBS	Bilateral *subthalamic nucleus stimulation* in PD
1997	FDA (USA)	DBS	Approval of the Medtronic 3382 DBS lead and the ITREL IPG for treatment of *tremor-dominant PD* (Paff et al. 2020)
1998	Nilsson et al. (Sweden)	LCIG	*Long-term intraduodenal infusion* of a water-based levodopa–carbidopa dispersion in very advanced PD (portable pump and *gastrostomy*)
1999	Chiesi farmaceutici S.p.A (Italy)	CSAI	Apofin™ approval (apomorphine, solution for infusion)^a^
2000	Aguettant (France)	CSAI	Apokinon™ approval (apomorphine, solution for infusion)^b^
2002	FDA (USA)	DBS	Approval of the Medtronic 3382 DBS lead and the ITREL IPG for treatment of *bradykinesia and rigidity* PD (Paff et al. 2020)
		DBS	Approval of Medtronic’s Activa system for *GPi DBS* for PD
2004	Britannia Pharmaceuticals Limited (UK)	CSAI	Apo-Go™ approval (apomorphine, solution for infusion)
2004	Sweden	LCIG	First country to approve Duodopa™, followed in 2005 by all 25 European Union Member States^c^
2008	–	DBS	First DBS *rechargeable* implantable pulse generator (Paff et al. 2020)
2013	Schuepbach et al. (international study group)	DBS	*EARLY-STIM study:* randomised trial, STN-DBS for PD with early motor complications
2015	Martinez-Martin et al. (International study group)	LCIG, CSAI	*Euroinf study:* multicentre comparative observational study of CSAI and LCIG
2015	FDA (USA)	LCIG	Approval of *carbidopa and levodopa enteral suspension* for the treatment of motor fluctuations in patients with advanced PD^d^
2016	FDA (USA)	DBS	Approval of Medtronic DBS for PD patients with *recent onset of motor complications* (following the EARLY-STIM study)^e^
2017	Senek et al. (Sweden)	LECIG	*Levodopa–entacapone–carbidopa intestinal gel* in PD (LECIG; LECIGon; LobSor Pharmaceuticals AB, Knivsta, Sweden)
2018	Lobsor Pharmaceuticals AB (Sweden)	LECIG	*Approval of Lecigon*™ (LECIG) by the Swedish Medical Products Agency (followed by Denmark, Finland and Norway in 2019)^f^
2018	Antonini et al. (international study group)	DBS, LCIG and CSAI	*5–2–1 criteria* to identify patients progressing to advanced PD and suitable for DAT (multi-country Delphi-panel approach)
2018	Katzenschlager et al. (International study group)	CSAI	*TOLEDO study:* randomised, placebo-controlled, double-blind, multicentre trial of CSAI for PD patients with persistent motor fluctuations
2019	Dafsari et al. (international study group)	DBS, LCIG and CSAI	*Euroinf 2 study*: real-life observational report of clinical efficacy of STN-DBS, CSAI and LCIG on quality of life, motor, and nonmotor symptoms in PD
2020	FDA (USA)	DBS	Approval of Percept™ PC with BrainSense™ technology for PD patients (neurostimulation system *recording brain signals while delivering therapy*)^g^
2020	Lobsor Pharmaceuticals AB (Sweden)	LECIG	Approval of Lecigon™ in Germany, the Netherlands, Belgium, Austria, Slovenia and Romania through the in European Mutual Recognition Procedure (MRP)

*CSAI* continuous subcutaneous apomorphine infusion, *DAT* device-aided therapies, *FDA* Food and Drug Administration, *GPi* globus pallidus internus, *LCIG* levodopa/carbidopa intestinal gel, *LECIG* levodopa–entacapone–carbidopa intestinal gel, *PD* Parkinson’s disease, *STN* subthalamic nucleus, *UK* United Kingdom, *USA* United States of America, *VIM* nucleus ventralis intermedius

^a^Ministerio della Sanita, Italia. Accessed February 24, 2023. https://www.gazzettaufficiale.it/atto/serie_generale/caricaDettaglioAtto/originario?atto.dataPubblicazioneGazzetta=1999-10-11&atto.codiceRedazionale=099A8418&elenco30giorni=false

^b^Haute Autorité de Santé (HAS), France. Accessed February 22, 2023. https://www.has-sante.fr/jcms/c_2740751/fr/apokinon-apomorphine

^c^EU/3/01/035: Orphan designation for the treatment of advanced idiopathic Parkinson's disease with severe motor fluctuations. Accessed February 22, 2023. https://www.ema.europa.eu/en/medicines/human/orphan-designations/eu301035

^d^AbbVie Announces U.S. FDA Approval of DUOPA™ (carbidopa and levodopa) Enteral Suspension for the Treatment of Motor Fluctuations in Patients with Advanced Parkinson's Disease. January 12, 2015. Accessed February 22, 2023. https://news.abbvie.com/news/abbvie-announces-us-fda-approval-duopa-carbidopa-and-levodopa-enteral-suspension-for-treatment-motor-fluctuations-in-patients-with-advanced-parkinsons-disease.htm

^e^FDA Approves Medtronic Deep Brain Stimulation for People with Parkinson's Disease with Recent Onset of Motor Complications. February 17, 2016. Accessed February 22, 2023. https://news.medtronic.com/2016-02-17-FDA-Approves-Medtronic-Deep-Brain-Stimulation-for-People-with-Parkinsons-Disease-with-Recent-Onset-of-Motor-Complications

^f^Lecigon development status. Accessed February 24, 2023. https://www.lobsor.com/lecigon-development-status/#

^g^FDA Approves “New Era” DBS Device. July 7, 2020. Accessed February 22, 2023. https://news.medtronic.com/fda-approval-percept

New formulations and devices, allowing the continuous *subcutaneous* infusion of levodopa, are getting close to entering the therapeutic armamentarium of PD specialists: ND0612 (Ramot et al. 2017; Olanow et al. 2021; Giladi et al. 2021; Poewe et al. 2021; LeWitt et al. 2022) and ABBV-951, a 24-h/day continuous subcutaneous infusion of a soluble levodopa/carbidopa phosphate prodrug combination (Rosebraugh et al. 2021a, b, 2022a, b; Soileau et al. 2022).

When faced with so many options, how to make a choice for a given patient? In other terms, and as worded by Nick Barber: “*what constitutes good prescribing?*” (Barber 1995). Patients may be suitable for a single DAT only (due to contraindication) but, most of the time, they face two or more choices (Volkmann et al. 2013). The Barber’s model encompasses four critical aims of drug prescribing: maximising effectiveness, minimising risks, respecting patient’s choice, and minimising costs (the latter being from both the healthcare system and patient’s perspective). The same considerations apply to medical devices, hence to DAT in PD. This viewpoint, therefore, aims to be thought-provoking and to assist clinicians in moving through the process of DAT selection, by including in their decision algorithm topics with which they are not necessarily familiar: their own biases, patient perspective, ethical concerns, and the current unknowns surrounding PD prognosis and DAT-related long-term side effects for a given patient.

## When to start thinking about DAT in advanced PD? On what grounds?

Although DAT are increasingly being offered earlier in the disease course in many expert centres (Schuepbach et al. 2013; Fernández-Pajarín et al. 2022), their classical indication remains advanced PD. Despite its wide use, the term “advanced PD” is still controversial, ambiguous, and rather subjective (Antonini et al. 2018; Fasano et al. 2019). It is largely defined by the emergence of dopaminergic motor and nonmotor complications leading to functional decline (worsening of quality of life, reduced independence in daily life activities), and by a reduction of response to conventional optimised oral therapy (Titova et al. 2017; Sesar et al. 2021). For non-PD specialists, identifying patients progressing to this stage, and, therefore, suitable for DAT, can be quite challenging (Luquin et al. 2017; Williams et al. 2017; Antonini et al. 2018; Fasano et al. 2019). Several attempts have, therefore, been made to reach consensus on the key factors for diagnosing advanced PD. In 2015, the NAVIGATE-PD program offered a collective physician perspective on DAT initiation (Odin et al. 2015). It was followed by the Spanish CEPA-study and the validation of a neurologist-based questionnaire, aiming at quickly identifying patients with advanced PD in the clinical setting (Luquin et al. 2017; Martinez-Martin et al. 2018). The international dissemination of this screening tool does not appear to be successful so far, probably because clinical key indicators of a transition to advanced PD were subsequently defined by a multi-country Delphi-panel involving PD specialists from 10 European countries (Antonini et al. 2018). The abbreviated version, known as the “*5–2–1 criteria*” (≥ five-times daily oral levodopa, ≥ two daily hours with ‘Off’ symptoms or ≥ one daily hour with troublesome dyskinesia) was launched in 2018 (Antonini et al. 2018), and has since been recognised as an objective, relevant and reliable tool, suitable for clinical practice (Santos-Garcia et al. 2020; Aldred et al. 2020; Malaty et al. 2022; Stefani et al. 2022; Antonini et al. 2022a). Nevertheless, the OBSERVE-PD study showed that this tool is not yet fully integrated into standard practice in many countries, as more than half of the patients identified by physicians as “non-advanced PD” actually met the 5–2–1 criteria (Fasano et al. 2022). Another screening tool, known as the MANAGE-PD tool (*Making Informed Decisions to Aid Timely Management of Parkinson’s Disease*) was recently designed by an international panel of PD experts (Antonini et al. 2021). Sound methodological questions regarding its development were, however, raised (Moes et al. 2022), and its relevance and accuracy still need to be assessed in the “real world” clinical practice. To date, identification of advanced PD patients, therefore, remains a challenge in many clinical settings.

Identifying patients with advanced PD is a critical but insufficient step. As highlighted by the OBSERVE-PD study (Fasano et al. 2019, 2022), apart from a few exceptions (Takáts et al. 2020; Evans et al. 2021; Möller et al. 2021), only a fraction of the patients deemed eligible for a DAT were initiated (Table 2).

**Table 2:** DAT-eligible patients and ongoing DAT in the OBSERVE-PD and PARADISE studies: insights into international heterogeneity

References	Country	Proportions of ongoing DAT in eligible advanced PD patients	Most used DAT
Fasano et al. (2019) (OBSERVE-PD)	18 countries	43.6% (DBS, CSAI or LCIG)	DBS (57%)
Takáts et al. (2020) (OBSERVE-PD)	Hungary	75% (unspecified)	Unspecified
Martínez-Castrillo et al. (2021) (PARADISE)	Spain	15.2% (DBS, CSAI, LCIG)	Unspecified
Szasz et al. (2021) (OBSERVE-PD)	Romania	45.7% (DBS, LCIG)	Unspecified
Evans et al. (2021) (OBSERVE-PD)	Australia	68% (DBS, CSAI, LCIG)	DBS
Möller et al. (2021) (OBSERVE-PD)	Switzerland	79% (DBS, CSAI, LCIG)	DBS
Stefani et al. (2022) (OBSERVE-PD)	Italy	41% (DBS, LCIG)	DBS
Pedrosa et al. (2022) (OBSERVE-PD)	Germany	40.8% (unspecified)	Unspecified

*CSAI* continuous subcutaneous apomorphine infusion, *DAT* device-aided therapies, *LCIG* levodopa/carbidopa intestinal gel, *PD* Parkinson’s disease

Most advanced PD patients are, therefore, chronically treated in a suboptimal way, because (1) they were either never referred to a PD specialist, (2) they were wrongly labelled as “non-advanced PD”, or (3) they were eligible but delaying or on a waiting list for DAT initiation (Fasano et al. 2019; Szasz et al. 2021; Stefani et al. 2022; Pedrosa et al. 2022). In 2017, one study estimated that only 10–15% of patients eligible for DBS were referred to specialised centres (Lange et al. 2017).

Theoretically, when faced with refractory motor and nonmotor fluctuations relevant to quality of life and activities of daily living, patients with advanced PD should be offered counselling for DAT to evaluate the risk–benefit ratio of an individual patient to undergo DAT. The real-world practice is far from these ideal. Factors accounting for these discrepancies need to be acknowledged and studied, whether they are linked to clinicians, patients and/or health systems.

## Looking at worldwide prescription patterns of DAT: does every patient with advanced PD has the same treatment opportunities?

Despite general guidelines issued by expert consensus or scientific international societies—including for DAT prescribing in PD (Hilker et al. 2011; Trenkwalder et al. 2015; Fabbri et al. 2018; Dijk et al. 2020), therapeutic approaches and prescription patterns differ internationally, but also nationally (Kalilani et al. 2019; Bruno et al. 2022).

### Worldwide use and repartition of DAT

Not all DAT are approved nor *realistically* available worldwide, due to financial limitations (including DAT costs and reimbursement issues), resource capacity and local expertise (Volkmann et al. 2013; Szaz et al. 2019; Henriksen et al. 2020; Bhidayasiri et al. 2020; Cramer et al. 2022). Apart from the OBSERVE-PD cohort (Fasano et al. 2019), data regarding national use and repartition of DAT are scarce (Ezat et al. 2017; Richter et al. 2019; Henriksen et al. 2020; Nordin et al. 2021; Thaler et al. 2022), particularly outside of Europe (Tables 2 and 3).

**Table 3:** Published national use and repartition of DAT

References	Country/time frame	Total number of DAT	DBS	CSAI	LCIG
Ezat et al. (2017)	Norway (2009–2013)	262	**146**	Not reported	116
Richter et al. (2019)	Germany 2010	Unspecified	**341**	130 initial setups 132 monitoring	81 initial setups 126 monitoring
	Germany 2017	Unspecified	**576**	194 initial setups (+ 49%) 233 monitoring (+ 77%)	159 initial setups (+ 96%) 261 monitoring (+ 107%)
Henriksen et al. (2020)	Denmark (2008–2016)	612	211	161 (CSAI + pen)	**283**
Nordin et al. (2021)	Sweden (2009–2019)	500 (implementations)	**225**	95	180
Thaler et al. (2022)	Israel (2009–2019)	161	**76**	23	62

*CSAI* continuous subcutaneous apomorphine infusion, *DAT* device-aided therapies, *LCIG* levodopa/carbidopa intestinal gel, *PD* Parkinson’s disease

DBS appears to be the oldest DAT available in many countries (~ 30 years) and the most prescribed (except in Denmark) with more than 150,000 implants worldwide (Henriksen 2020; Montemayor et al. 2022). Infusion therapies are less commonly used: within the OBSERVE-PD cohort, 39% of patients used LCIG, and only 8% were receiving CSAI (Fasano et al. 2019). The same ranking, with CSAI as the least used DAT, is found in most countries (Ezat et al. 2017; Richter et al. 2019; Henriksen et al. 2020; Nordin et al. 2021; Thaler et al. 2022). Of note, CSAI remains unavailable in many countries, including the United States of America^1^ and Japan (Auffret et al. 2018; Fasano et al. 2022; Fujioka et al. 2023). Although specialised clinical settings (including access to neurosurgery/gastroenterologist) are needed for DBS and LCIG/LECIG implementation (Richter et al. 2019; Henriksen et al. 2020), it is not the case for CSAI, which is considered as the easiest DAT to implement (Fasano et al. 2020). This striking lack of access to CSAI is, therefore, concerning, given its ease of initiation (minimally invasive, completely reversible, and no need of any kind of surgery), its strongly established efficacy on PD motor and nonmotor symptoms, and its good safety profile (Auffret et al. 2018; Katzenschlager et al. 2018, 2021; De Cock et al. 2022). LECIG has still limited data regarding its rate of implementation and repartition at the time of our writing, being a very recent addition to DAT therapies (Nyholm and Jost 2022).

Globally, there has been an increase in the use of DAT over the past decades (Richter et al. 2019; Henriksen et al. 2020; Norlin et al. 2021; Cramer et al. 2022), consistent with the overall increase in PD cases (Richter et al. 2019; Ou et al. 2021). However, the acute and long-term effects of the Covid-19 pandemic on this trend need to be determined. On one hand, the crisis created opportunities in the remote management of PD patients (Abate et al. 2020; Fasano et al. 2020; Roszmann et al. 2022) and even bolstered outpatient initiation of CSAI in France (Zagnoli et al. 2023). On the other hand, it led to acute, severe, and lasting disruptions and/or delays in DAT initiation requiring scheduled hospitalisations and/or surgeries (Fasano et al. 2020; Richter et al. 2021; Roszmann et al. 2022), as well as patient education (Roszmann et al. 2022). In addition, patients already receiving a DAT were also differently impacted by the pandemic, as demonstrated by an Italian study (Montanaro et al. 2022). PD patients treated with either DBS or LCIG during the lockdown experienced psychological distress, related to the fear of device dysfunction (and subsequent difficulties of obtaining adequate and rapid healthcare assistance), or the risk of a caregiver Covid-19 infection (Montanaro et al. 2022).

### Documented disparities in accessing DAT

Addressing global disparities in PD has been recently defined as a World Health Organization priority (Schiess et al. 2022). Unfortunately, there are known racial, gender and socioeconomic disparities in the general care of PD patients, even within a single healthcare system (Dahodwala et al. 2009; Willis et al. 2011; Henriksen et al., 2020; Nwabuobi et al. 2021; Subramanian et al. 2022). Yet, disparities in accessing DAT have been seldomly studied, apart from DBS (Crispo et al. 2020; Jost et al. 2022), for which most of the studies were led in the USA (Willis et al. 2014; Chan et al. 2014; Shpiner et al. 2019; Shirane et al. 2020; Watanabe et al. 2022). For DBS, concerning disparities regarding race (Willis et al. 2014; Chan et al. 2014; Shirane et al. 2020; Watanabe et al., 2022; Cramer et al. 2022), gender (Willis et al. 2014; Hariz et al. 2011; Shpiner et al. 2019; Shirane et al. 2020; Henriksen et al., 2020; Cramer et al. 2022; Watanabe et al. 2022; Jost et al. 2022), socioeconomic status (Willis et al. 2014), as well as insurance availability and type (Chan et al. 2014; Shpiner et al. 2019; Cramer et al. 2022) are consistently reported. White men are more likely to be referred and undergo DBS compared to women or their non–Caucasian counterparts (Willis et al. 2014; Chan et al. 2014; Shirane et al. 2020; Watanabe et al. 2022; Cramer et al. 2022; Deshpande et al. 2022; Jost et al. 2022). Moreover, greater disease severity and disability at the time of DBS referral is more common for women and non-Caucasian patients (Hariz et al. 2003; Shirane et al. 2020; Cramer et al. 2022; Jost et al. 2022), which can significantly reduce the window of opportunity to initiate surgery. Despite similar indications for all DAT, patients who are initiated on infusion therapies are older than those undergoing DBS, and more likely female (Richter et al. 2019).

All the above, therefore, question the “real-world” equal opportunity for PD patients, even within a single health system, and hint at differences in access to care, referral pattern biases (timing and frequency), physician biases (unconscious/implicit or conscious/explicit bias), patients’ preferences and health-seeking behaviour (Shirane et al. 2020; Crsipo et al. 2020; Cramer et al. 2022).

### Referral and access to PD specialists

Worldwide, physicians have been largely dichotomised into generalists and specialists (Swarztrauber and Vickrey 2004). Perceived as difficult by both medical students and physicians (Flanagan et al. 2007; Zinchuk et al. 2010), neurology has the same dichotomy, adding another layer of complexity (general neurologists and movement disorders specialists). Studies have shown differences in primary care physicians’ and neurologists’ preferences for involving a specialist in the care of patients with neurological conditions (Swarztrauber et al. 2002), as well as disagreement for the extent of specialty involvement in patients’ evaluation and management (Swarztrauber and Vickrey 2004). To be considered eligible for any DAT, patients must be referred to a neurologist, and preferably to a movement disorders specialist, who will assess whether they are suffering from advanced PD. The lack of referral has been consistently reported as a major impediment, notably for DBS, in North America, Asia and Europe (Henriksen et al. 2020; Zhang et al. 2020). Notably, women and minorities obtain neurologist care less often than white men (Willis et al. 2011), whereas early referral to a movement disorder specialist is important to maintain satisfactory levels of quality of life and ensure access to DAT (Williams et al. 2017).

Lack of awareness or knowledge on PD, and misjudgement of the need for referral among primary care physicians (or even general neurologists) are, therefore, a potentially insurmountable obstacle at the very first level of the care pathway (Swarztrauber et al. 2002; Swarztrauber and Vickrey 2004; Li et al. 2014; Ahlskog et al. 2020; Zhang et al. 2020).

## Do clinician biases exist when selecting DAT in advanced PD?

Available DAT are all indicated for advanced PD and show efficacy in treating both motor and nonmotor symptoms (Timpka et al. 2017; Dafsari et al. 2019; Deuschl et al. 2022). However, as previously highlighted, there are significant disparities in their repartition and prescription patterns, suggesting local habits and/or individual preferences (Carron et al. 2011; Richter et al. 2019). No established standard referral criteria, including timing or cut-off of improvement from medical management before proceeding with DAT, are used across providers (Cabrera et al. , 2019, 2021a; Marsili et al. 2021), paving the way to physician biases-related disparities (Hariz et al. 2003; Willis et al. 2014; Chan et al. 2014; Cabrera et al. 2019; Shirane et al. 2020; Watanabe et al. 2022; Cramer et al. 2022; Deshpande et al. 2022; Jost et al. 2022).

In the era of evidence-based medicine, clinical decision making involves the use of evidence, and encompasses both clinical expertise and the needs and wishes of individual patients (Bate et al. 2012). However, like the general population, health care providers (including neurologists and nurses) are faced with cognitive and affective, implicit and explicit biases, including racial and gender biases (Ryn et al. 2011; Lilienfeld and Lynn 2014; Marcum et al. 2017; Featherston et al. 2020; Tolsa et al. 2022; Thirsk et al. 2022). Numerous cognitive biases exist in clinical practice, particularly when using cognitive short-cutting (Croskerry 2002; Dobler et al. 2019). They need to be acknowledged as they influence clinicians’ behaviour and can seriously impact the quality, consistency and accuracy of clinical decision making, hence care delivery and patient’s outcome (Croskerry 2002; Bate et al. 2012; Klocko 2016; Featherston et al. 2020; Thirsk et al. 2022). They include *anchoring bias* (“undue emphasis given to an early salient feature during a consultation”), *ascertainment bias* (thinking influenced and shaped by prior expectations, like gender bias and stereotyping), *availability bias* (“recent experience dominates evidence”), *Bandwagon effect* (“*We do it this way here*”), *confirmation bias* (looking for supporting evidence rather than seeking information ruling it out), *omission bias* (tendency towards inaction, reluctance to treat) or *playing the odds* (opposite of the “rule out the worst case” scenario), *framing bias* (reaction to a choice varies depending on its presentation, for instance, as a loss or as a gain), *Sutton’s slip* (going for the obvious), *Gambler’s fallacy* (law of averages, sequence effect: “tendency to think that a run of diagnoses means the sequence cannot continue, rather than taking each case on its merits”), *search satisficing* (premature closure, or to stop investigating after having found one diagnosis, hence other co-existing conditions are not detected), *vertical line failure* (thinking in silos, or inside the box), *triage cueing* (“to create bias at the initiation of triage that then influences the ultimate choice of patient management”), *blind spot bias* (“*Other people are susceptible to these biases but I am not*”), *visceral bias* (emotional involvement), and *illusory correlation/superstition* (seeing a causal relationship between conditions, events or actions when there is none) (Croskerry 2002; Bate et al. 2012; Klocko 2016; Dobler et al. 2019). Emotional biases encompass *personal values* (anticipation of patient’s behaviour based on own values), *negative experience* (recollection of negative events) and *cultural bias* (judging exclusively from own cultural reference system) (Tolsa et al. 2022). Several clinicians related factors are, therefore, to be considered, encompassing the entire care pathway, from patient identification and referral to DAT selection, initiation, and follow-ups.

### Unfamiliarity or lack of personal experience

Not all neurologists have had personal experience with the implementation, management, and follow-up of the four existing DAT (Lange et al. 2017; Burack et al. 2018; Henriksen et al. 2020), even though a recent survey in Japan suggested that experience with DATs did not influence the directions of neurologist’s preferences (Fujioka et al. 2023).

Some consider themselves incompetent to determine whether a PD patient would be eligible for any DAT (Moes et al. 2022), notably due to limited knowledge about selection criteria (Lange et al. 2017). Knowledge about new indications or shift in DAT timing can also be limited (Cabrera et al. , 2019, 2021a), and uptake on new treatment guidelines has been shown to be slow, one explanation being the lack of neurologists with sufficient DAT experience (Norlin et al. 2021) or, more broadly, attachment to clinical experience, the latter possibly being related to the Bandwagon effect (Bate et al. 2012; Klocko 2016; Tolsa et al. 2022).

### Neurologists’ preferences and attitudes

Neurologists’ preferences for DAT in advanced PD have only been recently surveyed in Japan, (Fujioka et al. 2023). Based on hypothetical decision-making, treatment without the need for surgery (under development continuous subcutaneous infusion of levodopa–carbidopa) was strongly preferred, regardless of its need for frequent management, over DAT requiring surgery, namely LCIG and DBS (Fujioka et al. 2023). The findings of this study are thought-provoking, though they may not transfer to other countries, given that CSAI and LECIG are currently unavailable in Japan.

Contrary to infusion therapies, neurologists’ knowledge and attitude towards DBS and its timing has been investigated (Shih and Tarsy 2011, 2019, 2021a; Li et al. 2014; Cabrera et al. ). Significant differences between movement disorders specialists and non-specialists regarding medication and use of DBS in advanced PD are found (Shih and Tarsy 2011). Knowledge about DBS for movement disorders has been investigated in young neurologists from Egypt, as well as general neurologists from China, and deemed limited in both cases (Li et al. 2014; El-Jaafary et al. 2021).

More studies are needed to better understand neurologists’ preferences worldwide and how they transfer to clinical practice.

### Pitfalls of clinical trials

Clinical trials are an essential part of today’s evidence-based medicine. However, patients with advanced PD encountered in real-world practice often differ from those participating in clinical trials (Volkmann et al. 2013; Burack et al. 2018). This can lead to inadequate translation of study results into clinical practice (i.e. dose adjustment, severity of motor and nonmotor symptoms, side effects, degrees of improvement). In the BALANCE study, clinical practice regarding LCIG has been found to differ from the available evidence on best use, with delaying treatment initiation to elderly and more advanced patients, which led to higher rates of treatment-emergent adverse effects and inferior quality of life outcomes (Weiss et al. 2022).

Similarly, until the publication of the TOLEDO study, the only available data regarding CSAI were coming from observational or retrospective cohorts (Katzenschlager et al. 2018, 2021). As a result, these data coming from “real-word settings” were often criticised and/or disregarded in the scientific and medical literature, despite their relevance in clinical practice (Antonini et al. 2022b). On the contrary, STN-DBS has been extensively studied (Deuschl et al. 2022), and the abundance of scientific and medical literature may play in favour of its use.

### Medical myths and misconceptions

Over the years, various myths and misconceptions about neurology (neurophobia), PD and its treatment (i.e. levodopa phobia) have spread and flourished in the medical community, including among neurologists (Espay and Lang 2017; Ahlskog et al. 2020). These misconceptions also extend to DAT (Table 4). For instance, the need to (self-)inject has long been associated to a general perception of a “needle phobia”, with patients consequently being unwilling to use CSAI, but physicians may overestimate its extent among PD patients actually experiencing fluctuations (Imamovic et al. 2021).

**Table 4:** Concerns, bias, myths, and misconceptions potentially affecting DAT selection among clinicians and patients

DAT	Clinicians	Patients
All DAT	• Gender and/or racial bias (referral and DAT choice) • Neurophobia (Menken 2002; Zinchuk et al. 2010; Flanagan et al. 2007) • Unfamiliarity/lack of personal experience (availability/approval) • Exposure to local practice (Bandwagon effect) • Negative experience emotional bias: recollection of a poor clinical outcome or negative reactions to a previous decision (Tolsa et al. 2022)	• Cultural biases or beliefs (Chan et al. 2014) • Medical mistrust in ethnic groups consecutive to repeated past mistreatment (Cramer et al. 2022) • Being labelled as “advanced PD” is pejorative and suggestive of irreversibility (Williams et al. 2017; Fasano et al. 2022) • Lack of information/education • Medical decision capacity, potentially influenced by PD characteristics and/or treatment (Dymek et al. 2001; Cranston 2001; Martin et al. 2008; Trachsel et al., 2014; Abu Snineh et al. 2017; Sokol et al. 2019; Alfonso et al. 2022) • Personal risk tolerance (risk aversion vs impulsive behaviours), potentially influenced by PD characteristics and/or treatment (Alfonso et al. 2022) • Preferences for involvement in healthcare decision making (Zizzo et al. 2017)
DBS	• Not appropriate for older patients (Mariani 2021) • Gender and/or racial referral bias (Willis et al. 2014; Chan et al. 2014; Shirane et al. 2020; Watanabe et al. 2022; Cramer et al. 2022; Jost et al. 2022) • Overestimation of patient’s reluctance to undergo DBS (Lange et al. 2017) • Marketing exposure (Richter et al. 2019) • Overly optimistic portrayal (social media exposure, direct and indirect marketing exposure) and under-estimation/disregard of side effects (Smailhodzic et al. 2016; Richter et al. 2019; Al Busaidi and Alamri, 2020; Braczynski et al. 2021) • Undesirable non-stimulation-dependent effects (Blume et al. 2017; Pugh 2019)	• A technical fix/overly optimistic portrayal on social media (Smailhodzic et al. 2016; Al Busaidi and Alamri 2020; Braczynski et al. 2021) • Preference for innovative technology • Fear of neurosurgery, particularly awake surgery, and of complications (Hamberg and Hariz 2014; LaHue et al. 2017; Shpiner et al. 2019; Jost et al. 2022; Vinke et al. 2022) • Concerns over technical problems (Montemayor et al. 2022; Alfonso et al. 2022) • Concerns about DBS affecting relationships (Alfonso et al. 2022; Montemayor et al. 2022) • Concerns about earlier use, including not having exhausted other non-invasive methods (Sperens et al. 2017; Cabrera et al. 2020; Montemayor et al. 2022; Alfonso et al. 2022) • Financial concerns (Das et al., 2021)
LCIG / LECIG	• “Last resort option” started to late (Weiss et al. 2022) • Lack of personal experience • “Levodopa anxiety/phobia”: “levodopa is toxic”, “levodopa stops working after a period of time”, “levodopa induces dyskinesia” (Espay and Lang 2017; Titova et al. 2018; Ahlskog et al. 2020) • Not appropriate for older patients (Mariani 2021)	• “Last resort option” (Weiss et al. 2022): suggestive of irreversibility • “Levodopa anxiety/phobia” (Titova et al. 2018), including “levodopa stops working after a period of time” (Salinas et al., 2020) • Lifestyle limitations and personalised social stigma due to device’s visibility (Volkmann et al. 2013) presence of a permanent gastrojejunostomy catheter, and need to carry a large drug pump (LCIG), requirement to carry a device and to learn additional procedure. (Aydemir et al. 2022)
CSAI	• Needle phobia (Imamovic et al. 2021) • Lack of personal experience • Cannot be initiated without antiemetics (Isaacson et al. 2023) • Dopamine agonist phobia (Rota et al. 2020) • Fear of side effects (vomiting, nodules) • Can only be initiated as an inpatient treatment (Castaño et al. 2007; Zagnoli et al. 2023) • Not appropriate for older patients (Mariani 2021) • Lack of “robust” trials, as defined by evidence-based medicine (Katzenschlager et al. 2018) • Unsafe in any patient with cognitive symptoms or hallucinations (Morgante et al. 2004; Borgemeester and van Laar 2017)	• Needle phobia (Titova et al. 2018)—need for frequent injections (Aydemir et al. 2022) • Association of apomorphine to morphine • Lifestyle limitations and personalised social stigma due to device’s visibility (Volkmann et al. 2013) requirement to carry a device and to learn additional procedure (Aydemir et al. 2022)

*CSAI* continuous subcutaneous apomorphine infusion, *DAT* device-aided therapies, *LCIG* levodopa/carbidopa intestinal gel, *PD* Parkinson’s disease

Social media are increasingly used by health care professionals and students for various reasons, including education or teaching purposes (Ventola 2014; Al Busaidi and Alamri 2020; Lynn 2022). Lack of content quality and reliability, as well as direct or indirect marketing exposures (sponsored content) on different platforms are matters of concerns in this regard (Ventola 2014; Gardner et al. 2019). Overly optimistic portrayals of DBS on social media have indeed been reported, particularly in YouTube videos (Gardner et al. 2019). These considerations may extend to other DATs, although this has not been studied yet.

## Patient preference, biases, and perspective: when and how do they come into play?

Patient-related demographics (age, race, sex), socioeconomic factors (educational background, insurance availability), personal experience (DAT exposure, including through social media and online communities), as well as educational influence of and trust in the therapy-applying clinician have all been shown to influence DAT access (referral), preference (surgery or infusion therapies), or acceptance (Smailhodzic et al. 2016; Kim et al. 2017; Shpiner et al. 2019; Richter et al. 2019; Montanaro et al. 2019; Cabrera et al. 2021a, b; Henriksen et al. 2020; Al Busaidi and Alamri 2020; Tripathi et al. 2020; Das et al. 2021; Braczynski et al. 2021).

Transitioning from oral drug administration to DAT is considered as an important step for patients (Fasano et al. 2022). Hence, fear of invasive treatments labelled as “advanced” therapies, excessive anxiety, lack of motivation, “need to have more time to decide”, fear of lifestyle limitations and personalised social stigma are common patients-related reasons for non-initiation (Volkmann et al. 2013; Burack et al. 2018; Stefani et al. 2022; Pedrosa et al. 2022). They may be underpinned by knowledge gaps and/or misconceptions (Table 4) about PD and therapeutic options (Lökk et al. 2011; Jitkritsadakul et al. 2017; Salinas et al. 2020). Cognitive biases (attentional, interpretation, and recall) are also prevalent in chronic illness and may influence patients’ motivation and decision making, hence health management (Savioni and Triberti 2020). Of note, PD patients have been shown to exhibit an *attributional* bias (cognitive bias, mistakenly attributing a situation to one cause) compared to controls, particularly when treated with DBS (Decombe et al. 2022).

PD patients exhibit different preference patterns when weighting treatment benefits and harms, focussing either on optimising the process of care, or controlling motor symptoms (Weernink et al. 2013). In addition, preferences for participation in decision making (how patients want to be involved in their own care) are known to vary in PD patients, depending on decision type, context, and relational factors (Zizzo et al. 2017). Broadly speaking, PD patients can (1) prefer to make the final decision, (2) opt for a shared choice (largely preferred in most cases), or (3) prefer to delegate final decisions to the physician (Zizzo et al. 2017). In all cases, however, they want to be informed of treatment options and involved in the deliberation (Zizzo et al. 2017). Careful assessment of individuals’ preferences on an ongoing basis and appropriate clinical guidance and education are, therefore, needed.

### Patient preferences for DAT in advanced PD

Patient preference is a significant part of the decision-making process (Carron et al. 2011; Volkmann et al. 2013; Richter et al. 2019). It is also critical for treatment adherence, as DAT initiation and management requires patient’s full cooperation (Carron et al. 2011; Volkmann et al. 2013; Richter et al. 2019). Patient preferences for DAT in advanced PD have received some attention in the recent years (Marshall et al. 2017; Aydemir et al. 2022), particularly for DBS (Shpiner et al. 2019; Das et al. 2021; Jost et al. 2022; Vinke et al. 2022; Alfonso et al. 2022; Montemayor et al. 2022), including preferences for its earlier use (Cabrera et al. 2020, b; Sperens et al. 2017; Alfonso et al. 2022; Montemayor et al. 2022).

Despite the high prevalence of DBS among DAT in real-world clinical practice, as previously highlighted, several studies show that patients generally view it as a secondary treatment option to medication (Marshall et al. 2017; Sperens et al. 2017; Cabrera et al. 2021b; Aydemir et al. 2022; Montemayor et al. 2022). In a web-based survey of American patients with advanced PD (*N* = 401), the idea of treatment delivery via an infusion pump (LCIG) was preferred over DBS (Marshall et al. 2017). In a Turkish survey, PD patients (*N* = 58) were more likely to decline STN-DBS and LCIG due to surgical concerns, while CSAI was declined due to the need of repeated injections (Aydemir et al. 2022). Disease severity and age also played a role in patient preference, with STN-DBS being preferred by younger, less severe patients, and CSAI by older patients with a longer disease duration (Aydemir et al. 2022). In this Turkish survey, LCIG was the least preferred treatment (Aydemir et al. 2022).

Again, contrary to infusion therapies, patients’ preference and attitude have been largely studied for DBS (Hamberg and Hariz 2014; Sperens et al. 2017; LaHue et al. 2017; Shpiner et al. 2019; Furlanetti et al. 2020; Cabrera et al. 2020, 2021b; Hauber et al. 2021; Montemayor et al. 2022; Vinke et al. 2022; Jost et al. 2022; Alfonso et al. 2022; Cramer et al. 2022). Three different approaches to DBS were identified among PD patients: “*taking own initiative*”, “*agreeing when offered*”, and “*hesitating and waiting*” (Hamberg and Hariz, 2014). When offered, female patients are more likely to decide against undergoing DBS, possibly due to “greater fear of surgery” (Jost et al. 2022) and/or “strong fear of complications” (Hamberg and Hariz 2014; Shpiner et al. 2019). However, women are more likely to undergo DBS when offered to be operated asleep (Vinke et al. 2022). These findings suggest that gender-related factors may be playing a role in the gender disparity in DBS (Shpiner et al. 2019), but whether this is due to a distinct PD profile (higher level of anxiety in women, Cerri et al. 2019) or implicit/explicit biases remains to be determined. Factors contributing to preference between asleep or awake surgery may include concerns or fear of being awake during neurosurgery, claustrophobia, anxiety, pain or discomfort during the procedure (stereotactic frame placement, surgery), comorbid pain conditions, severe off-medication symptoms, but also feeling self-conscious or being curious (LaHue et al. 2017). DBS perception and timing, assessed in a US cohort of patients with PD but without DBS (*N* = 285), showed differences in concerns regarding DBS safety, efficacy, and favourability comparing to medical management (Alfonso et al. 2022). Exposure to the reality of DBS, through PD organisation or associations, may also have a “deglamourizing” effect on patients, particularly regarding side effects, and influence their preference (Sperens et al. 2017). Patients’ attitudes on the early use of DBS appear to be mixed (Sperens et al. 2017; Cabrera et al. 2020; Montemayor et al. 2022), including in those who already benefited from DBS, and who would not necessarily have endorsed its implementation earlier in their own PD course (Cabrera et al. 2020). Patients’ tolerance for risk (worsening depression or anxiety, brain bleed or death) and willingness to wait for potential benefits of new devices also vary, and are related to patients age, ambulation, and prior neurostimulation experience (Hauber et al. 2021). Patients may also express preferences regarding DBS systems, notably on the battery life duration, rechargeability, and size (Furlanetti et al. 2020; Lee et al. 2022). When choosing between fixed-life or rechargeable battery, the size of the battery seems to be an important factor in *long-term satisfaction*, while being quite overlooked preoperatively (Furlanetti et al. 2020). Finally, patient preference for innovative technologies may differ between ethnic groups (Cramer et al. 2022).

### Unfamiliarity—lack of information

Lack of information and misconceptions are prevalent among PD patients (Li et al. 2014), as highlighted by the recent KnowPD study (Salinas et al. 2020). Moreover, only a small proportion of patients are informed about DAT options, particularly earlier in the course of the disease (Lökk et al. 2011).

To meet their information needs, patients frequently turn to online communities and social media (Chu and Jang 2022). For instance, questions regarding DBS, other patients’ experiences or choices relating to treatment, decision making on treatment options, and health coverage were frequently found in free-posting messages of a large online community of South Korean patients and family members (Chu and Jang 2022). Social media platforms are a readily accessible and ever-growing source of health-related information and medical education for patients and caregivers, despite contents of unverified origin (medical, health-related commercial entities, individual users?) and of extremely variable scientific quality, reliability, and accuracy (Smailhodzic et al. 2016; Kim et al. 2017; Al Busaidi and Alamri 2020; Tripathi et al. 2020; Braczynski et al. 2021). The consequences of social media use on patients have been carefully studied, and encompass improved self-management and control, enhanced psychological well-being, enhanced or diminished subjective well-being, addiction to social media, loss of privacy, and being targeted for promotion (Smailhodzic et al. 2016). The latter is of great concern, particularly as corporate interests are sometimes hidden behind seemingly genuine patients’ testimony YouTube videos (Gardner et al. 2019). A significant amount of DBS-related YouTube videos indeed offers over-optimistic portrayals, with dramatic “before and after” or “on/off” effects, without equally highlighting risks, thus contributing to the misleading myth of a “technological fix” and raising public expectations (Gilbert and Ovadia 2011; Gardner et al. 2019). Social media use also affects the relationship between patients and healthcare practitioners (Smailhodzic et al. 2016).

### The influence of PD on medical decision and treatment choice

The question of decision-making abilities (including decisional capacity, medical information processing, capacity to consent, and ability to understand informed consent) of (1) patients with advanced PD and potential cognitive impairment or fluctuations, impairment and/or adverse cognitive effects related to their PD treatment (impulsive cognitive disorders, apathy), and (2) younger-onset PD patients assessed for early-DBS eligibility (but more prone to risk-taking behaviour and impulse control disorders) is currently far from being sufficiently investigated (Dymek et al. 2001; Cranston 2001; Griffith et al. 2005; Martin et al. 2008; Eygelshoven et al. 2017; Sokol et al. 2019; Koerts et al. 2020; Alfonso et al. 2022).

In a Dutch study, no impairment in medical decision making was found in non-demented PD patients compared to healthy controls (Eygelshoven et al. 2017). However, the sample consisted of fairly young patients (mean age 60.9 years old), with rather early disease stages (mean Hoehn and Yahr stage 2, mean disease duration 5 years and mean Levodopa Equivalent Daily Dose 565 mg). Cognitive complaints in non-demented PD patients were found to impact their capacity to understand, appreciate and reason, and, therefore, to make a valid decision (Abu Snineh et al. 2017). While patients still express a choice, it does not necessarily mean that they fully understand the information presented to them and evaluate their congruence with their values and goals of care (Abu Snineh et al. 2017). Cognitive fluctuations are also a reality in PD (Trachsel et al. 2015). Finally, executive dysfunction may impair PD patient’s abilities to weigh different factors and to anticipate personal consequences of treatment decisions (Griffith et al. 2005). Impairment in decisional capacity increases as PD progresses (Griffith et al. 2005; Martin et al. 2008; Eygelshoven et al. 2017; Abu Snineh et al. 2017). Timing of patient’s information about DAT is, therefore, critical, and whether the patient’s cognitive capacity is already reduced needs to be carefully assessed before moving forward with DAT choice and initiation (Hug et al. 2021).

## Embracing prognostic uncertainty and unforeseeable outcomes: long-term safety and individual trajectories

The four ethical principles of healthcare encompass autonomy, justice, beneficence and non-maleficence, the latter referring to avoiding or preventing harm (Koerts et al. 2020). PD subtypes (Katz et al. 2015; Xu et al. 2018; Campbell et al. 2020), genetic background (Chan 2022), and unpredictable long-term side effects of DAT are elements which at present remain in the realm of uncertainty as to the patient’s individual response. Currently, there is limited knowledge on patient progression and DAT long-term outcomes. DAT are not risk-free and may pose ethical challenges (Hug et al. 2021).

Notably, DBS and its earlier use (i.e. briefly after the onset of the first dopaminergic response fluctuations) raise multiple questions regarding safety, as atypical parkinsonism can mimic early PD, dopaminergic treatment may influence risk-taking behaviour (impacting risk preference and/or assessment), unanticipated changes in personality, self, and relationships behaviour may emerge, and the risk of a floor effect or iatrogenic harms in the long run cannot be excluded (Schüpbach et al. 2006; Schuepbach et al. 2013; Cyron 2016; Kim and Jeon 2019; Thomson et al. 2020; Gilbert and Lancelot 2021). DBS may also be considered as an exclusion criterion in current and future clinical trials looking at disease-modifying treatment. It is the case for the AMBITIOUS study, a multicentre, randomised, double-blind, placebo-controlled clinical trial investigating whether the prolonged administration of ambroxol can change glucocerebrosidase (GBA) enzyme activity and alpha-synuclein levels in PD patients with GBA mutations.^2^ This aspect must be disclosed to patients, particularly when considering early-DBS.

Infusion therapies are not without adverse effects either. LCIG treatment requires long-term tube placement, necessitating a careful monitoring of the PEG-J tube, but also periodic tube replacement, exposing the patient to both material and procedural risks (Epstein et al. 2016; Yamashita et al. 2021).Without good skin management, CSAI-related cutaneous side effects jeopardise the long-term retention of this therapy and patients’ comfort and are one of the main reasons for its discontinuation (Olivola et al. 2019; Henriksen and Staines 2021).

Increasing evidence point out to a direct influence of PD subtypes on therapeutic response to DBS, and even mortality rate (Katz et al. 2015; Xu et al. 2018; Campbell et al. 2020). Similar studies have yet to be undertaken for infusion therapies.

There is very preliminary insight, on whether genetic background would affect DBS long-term outcome (Chan 2022). Most studies suffer from essential limited sample sizes needed for clinico-genetic association studies, and based on very small cohorts, LRRK2 and PRKN mutations carriers were more likely to enjoy good surgical outcomes. There is uncertainty on GBA carriers that may show more severe nonmotor and cognitive disease progression—one study reported that STN-DBS might deteriorate cognitive performance over what might be expected from disease progression (Chan 2022). However, all these rather hypothesis generating than confirmatory studies need independent confirmation including larger cohorts.

## “Only one answer” or “Choose all that apply”: is there really only one option that is better than all others for a given patient?

Advanced PD patients are often eligible for two or more DAT (Volkmann et al. 2013). Beyond the contraindications of each of the currently available DAT, one can question the mutually exclusive and competing approach often found in the literature (Carron et al. 2011). Is one DAT *truly better* than the others, and is that choice irrevocable? From our perspective, it is quite the opposite: the diversity of options allows a fine adjustment to each patient’s needs, considering disease course, burden, iatrogenic risk, and goals of care. It is, therefore, time to advocate for a dynamic approach, involving DAT switch and/or combination, as part of a continuum in the management of a chronic multisystem disorder (Table 5).

**Table 5:** Switching and combining device-aided therapies

Initial DAT	Switching DAT	Combining DAT
CSAI	CSAI to DBS CSAI to LCIG/LECIG	CSAI + DBS
LCIG	LCIG to LECIG LCIG to DBS	LCIG + DBS LCIG + night-time CSAI
LECIG	LECIG to DBS	LECIG + DBS
DBS	DBS to CSAI DBS to LCIG/LECIG	DBS + (night-time) CSAI DBS + LCIG/LECIG

*CSAI* continuous subcutaneous apomorphine infusion, *DAT* device-aided therapies, *LCIG* levodopa/carbidopa intestinal gel, *LECIG* levodopa–entacapone–carbidopa intestinal gel

### Switching between device-aided therapies: a sequential approach

Switches between DAT are frequent in clinical practice (Georgiev et al. 2022). Currently, all DAT are considered *reversible* (Volkmann et al. 2013). This, however, needs to be tempered for DBS, as undesirable non-stimulation-dependent effects may occur and deserve to be further investigated (Pugh 2019; Hug et al. 2021). White matter lesions, induced by brain surgery and electrode(s) trajectory (notably intersecting with caudate nuclei), may have deleterious effects on patient’s cognitive status (Witt et al. 2013; Blume et al. 2017). Nonetheless, if one DAT becomes unsuitable, patients have the option of trying another (Volkmann et al. 2013).

Most notably, PD patients on the waiting list for DBS frequently benefit from infusion therapies before surgery. Being minimally invasive, CSAI appears to be the DAT of choice in this case (Alegret et al. 2004; Fernández-Pajarín et al. 2021; Henriksen and Staines 2021; Georgiev et al. 2022) but LCIG can also be used (Georgiev et al. 2022). The infusion therapy is usually stopped after DBS initiation.

Confronted with the limitations of one approach, either because of adverse effects, DAT-related complications or symptoms resurgence (i.e. sleepiness with CSAI, infections with DBS, digestive complications with LCIG), patients can be switched from one DAT to another: CSAI to DBS (Varma et al. 2003; Kimber et al. 2017; Sesar et al. 2017; Olivola et al. 2019; Georgiev et al. 2022), CSAI to LCIG (Kimber et al. 2017; Georgiev et al. 2022), LCIG to DBS (van Poppelen et al. 2021) or DBS to infusion therapies (Sesar et al. 2019).

Improvements in the drug/device combination may also lead to a change, as recently evidenced by the addition of entacapone to the levodopa/carbidopa intestinal gel, and the subsequent switch from LCIG to LECIG (Senek et al. 2017; Öthman et al. 2021; Jost et al. 2023).

Within the next few years, switches to continuous subcutaneous levodopa infusion are expected.

### Combining device-aided therapies: a dual perspective

Combining DAT are not uncommon in clinical practice (Sesar et al. 2017, 2019; Fasano et al. 2019; Boura et al. 2021; Thaler et al. 2022; Georgiev et al. 2022). If studies rigorously (as defined by evidence-based medicine) assessing the efficacy of a dual therapy are currently lacking, retrospective cohorts and case series from different countries point out to improvements in fluctuations and quality of life in patients treated with a combination of surgery and infusion therapy (Sesar et al. 2017, 2019; Boura et al. 2021; Georgiev et al. 2022).

The increasing prevalence of DBS patients, and particularly of patients operated early in the disease course (Schuepbach et al. 2013), raises the probability of the need for combined therapies, as DBS does not prevent nor modify disease progression. In patients who previously benefited from DBS, but whose symptoms are inadequately controlled (persistent or reemergent fluctuations) or in case of DBS failure, CSAI (Sesar et al. 2017, 2019; Georgiev et al. 2022) or LCIG (Buhmann et al. 2017; El Kouzi et al. 2018; van Poppelen et al. 2021; Georgiev et al. 2022; Isaacson et al. 2022; Abu Al-Melh et al. 2023) can be initiated concomitantly, with an additional and complementary beneficial effect, even in advanced PD.

Similarly, but more rarely, adding DBS to LCIG treatment allows an improvement in motor fluctuations, but also a reduction in levodopa dose, of clear interest when patients suffer from dopaminergic side effects (Buhmann et al. 2017; Boura et al. 2021; van Poppelen et al. 2021).

Again, we may expect to see in the future combinations of DBS and continuous subcutaneous levodopa infusion.

## How can we ensure the most appropriate and personalised treatment for patients with advanced PD? A summary and looking at future perspectives

**Fig. 1:**
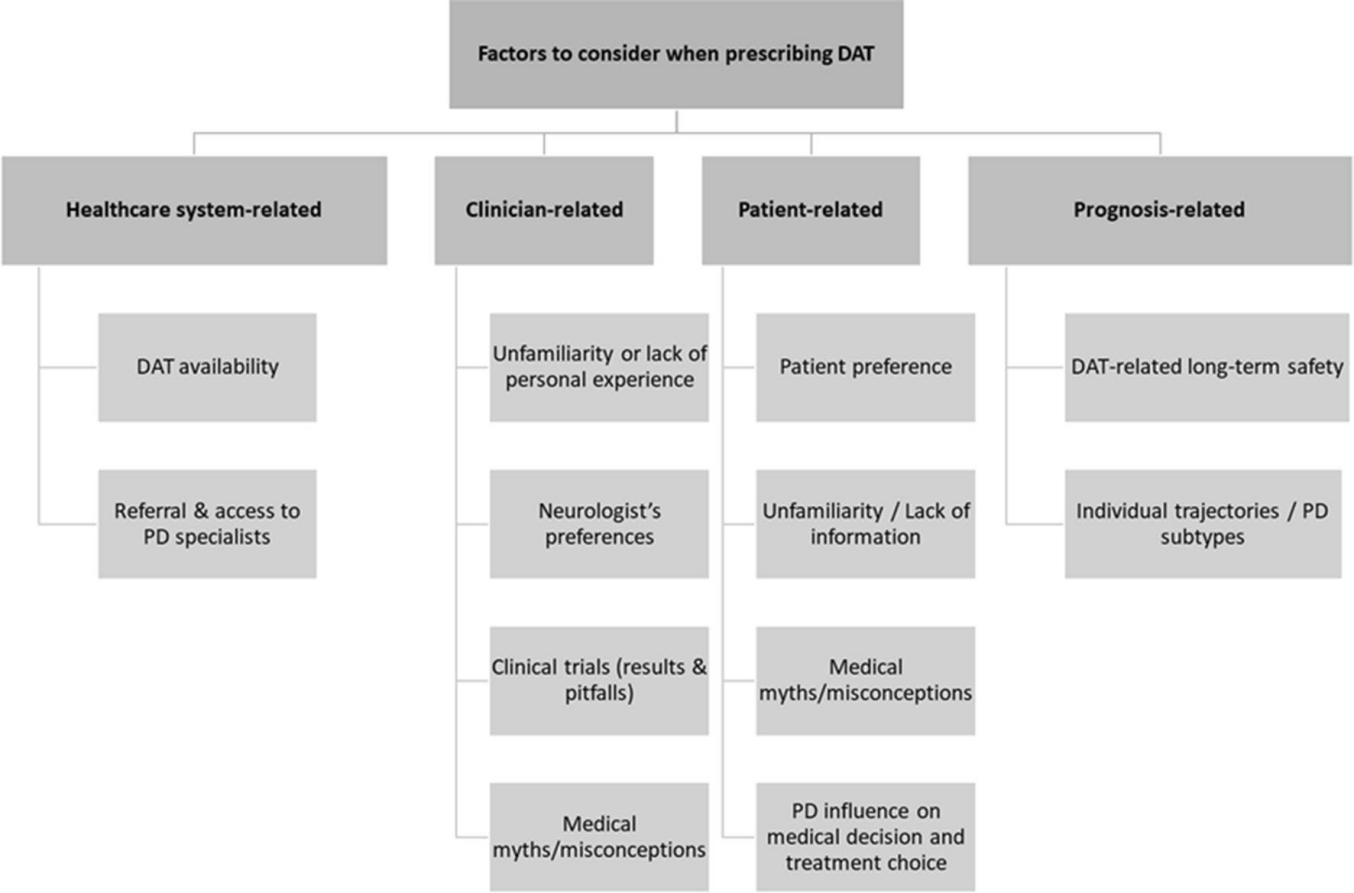
Factors to consider when prescribing device-aided therapies. *DAT* device-aided therapies,* LCIG* levodopa/carbidopa intestinal gel,* PD* Parkinson’s disease

As movement disorder specialists expect to see a rise in the number of PD patients needing DAT in the future (Marsili et al. 2021), persistent disparities need to be addressed (Cramer et al. 2022; Subramanian et al. 2022). Moving through the process of DAT selection can be complex (Fig. 1).

**Fig. 2:**
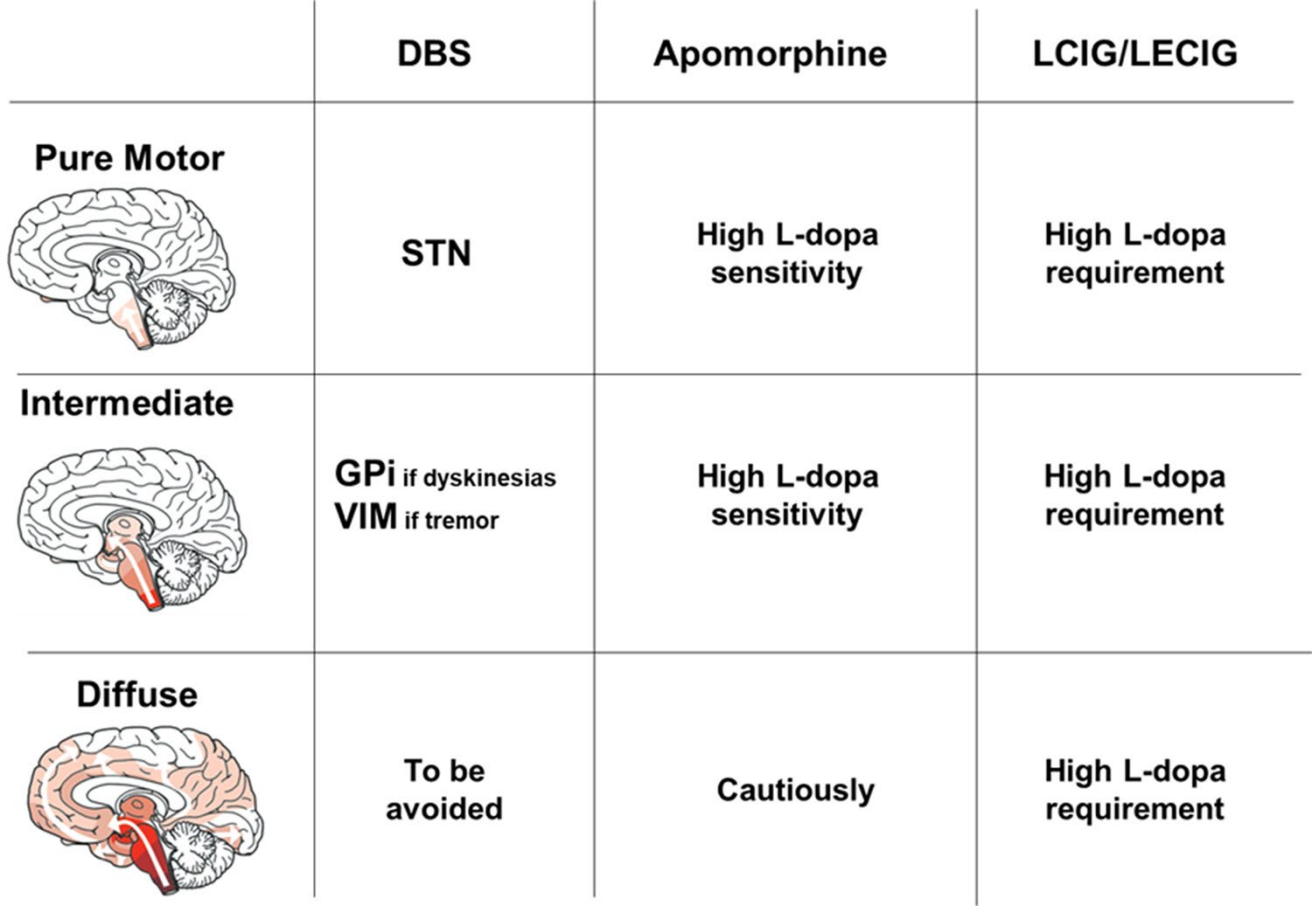
Which device-aided therapy for which patient? A pragmatic approach. DBS deep brain stimulation,* LCIG* L-dopa carbidopa intestinal gel,* LECIG* L-dopa entacapone carbidopa intestinal gel,* STN* subthalamic nucleus,* GPi* globus pallidus internalis,* VIM* thalamic ventral intermedialis nucleus, high L-dopa sensitivity: < 1000 mg L-dopa/day; High L-dopa requirement: > 1000 mg L-dopa/day

Although guidelines and recommendations have been regularly published and updated (see Fig. 2 for a pragmatic approach relevant to clinical practice, based on Putzke et al. 2003; Rouaud et al. 2010; Hilker et al. 2011; Fereshtehnejad et al. 2015; Trenkwalder et al. 2015; Bonenfant et al. 2017; Katzenschlager et al. 2018; Dafsari et al. 2019; Fabbri et al. 2018; Dijk et al. 2020), their pertinence regarding a specific patient’s situation can be questioned. Several challenges must be overcome, both in clinical practice and research, to improve patients’ identification (referral), eligibility (DAT approval and availability), DAT selection, initiation, and follow-up (cost, available resources).

Reducing disparities in health care (notably racial, gender and socioeconomic disparities) requires changing physician behaviour on a local and institutional level (Cramer et al. 2022): this can be achieved with medical training, and educational, real-time workplace strategies (Dobler et al. 2019; Cramer et al. 2022). Lengthy waiting times or financial restrictions must be fought at local, regional, national, and international level: it is now necessary for patient associations, medical teams, device companies and governments to work together and ensure that all PD patients have equitable access to DAT. Home initiation, titration and follow-up, with the help of telemedicine, can be resource-efficient, well-accepted and satisfactory for both patients and the clinical team (Willows et al. 2017; Zagnoli et al. 2023): these approaches need to be developed to ensure DAT access, particularly in areas where access to specialists can be challenging. Devices’ approval and availability should be a top priority in the list of actions to end global disparities in PD.

Research is needed to better understand what factors shape clinicians’ willingness to (not) refer PD patients for DAT information and assessment earlier in the disease course (Cabrera et al. , 2019, 2021a). Notably, studies exploring referral patterns among general practitioners and neurologists are needed to reach better informed and earlier referrals (Evans et al. 2021; Jost et al. 2022). Indeed, advance planning perspective must be given priority, owing to PD-related impairments in decisional capacity (Griffith et al. 2005; Hug et al. 2021). Early and multiple discussions are critical to identify patients ‘values and priorities, address knowledge gaps, build familiarity (and overcome potential negative prejudices) with available DAT options (Burack et al. 2018; Hug et al. 2021; Möller et al. 2021; Alfonso et al. 2022). Though time consuming, increased education regarding the risks and benefits of DAT, as well as community outreach, will allow patients and caregivers to make an informed decision as to the most appropriate therapy to their individual needs, and to move forward with implementation at the appropriate timing (Volkmann et al. 2013; Burack et al. 2018; Shpiner et al. 2019; Alfonso et al. 2022). Patient education should also involve critical assessment of healthcare-related YouTube videos (Tripathi et al. 2020). Recent efforts towards identifying PD patient-centred regulatory endpoints for medical devices have been undertaken with the FDA (Benz et al. 2021). Along with improving shared-decision processes, these initiatives should be pursued to ensure a patient-centred standard of care.

In addition, recent progresses have been made in identifying different PD sub-phenotypes through the analysis of brain connectivity (Yassine et al. 2022, 2023). In the near future, this innovative profiling may play a pivotal in the patients’ selection process, as a potential biomarker for individual trajectories.

Compared to DBS (Shih and Tarsy 2011; Lange et al. 2017), little information is available concerning the use of infusion therapies, as well as neurologist and patients attitudes towards them. Studies focussing on both physician and patient’s attitude, knowledge, and perspective towards LCIG, LECIG and CSAI are, therefore, urgently needed.

## Conclusion

The landscape is rapidly evolving in the therapeutic armamentarium of advanced PD, with the approval of LECIG, hopes for CSAI approval in the USA, and the forthcoming arrival of continuous subcutaneous levodopa infusion in clinical settings. Although clinical practices are heterogeneous and treatment individualisation mandatory, advance planning, ongoing education, and a multidisciplinary approach are advisable in all cases (Burack et al. 2018). New methods of initiation and titration (particularly at home) are likely to change the preferences of both patients and clinicians, and to improve accessibility (Zagnoli et al., 2023). Studies focussing on infusion therapies are urgently needed, as little information is available concerning neurologists’ and patients’ attitudes towards them, compared to DBS.

## Data Availability

Not applicable.

## Footnotes

1 Supernus provides regulatory update on SPN-830. News release. Supernus Pharmaceuticals, Inc. October 10, 2022. Accessed February 21st, 2023. https://ir.supernus.com/news-releases/news-release-details/supernus-provides-regulatory-update-spn-830.

2 https://clinicaltrials.gov/ct2/show/NCT05287503.
